# The Novel-miR-659/*SPP1* Interaction Regulates Fat Deposition in Castrated Male Pigs

**DOI:** 10.3390/ani12080944

**Published:** 2022-04-07

**Authors:** Lianmei Xiao, Qiao Xu, Ximing Liu, Shuheng Chan, Yabiao Luo, Shuaihan He, Meiying Fang

**Affiliations:** 1 Department of Animal Genetics and Breeding, National Engineering Laboratory for Animal Breeding, MOA Laboratory of Animal Genetics and Breeding, Beijing Key Laboratory for Animal Genetic Improvement, College of Animal Science and Technology, China Agricultural University, Beijing 100193, China; 17785245395@163.com (L.X.); xuqiao987@163.com (Q.X.); lximing2018@163.com (X.L.); 15380345581@163.com (S.C.); yabiaoluo2021@163.com (Y.L.); heshuaihan@cau.edu.cn (S.H.); 2 School of Life Sciences, University of Chinese Academy of Sciences, Beijing 100049, China; 3 Sanya Institute of China Agricultural University, Sanya 572025, China

**Keywords:** *SPP1*, castration, fat deposition, novel-miR-659

## Abstract

**Simple Summary:**

Castration is a standard method for eliminating boar taint in industrial hog production, but it also causes enormous fat accumulation in the carcass. Secreted phosphoprotein 1 (*SPP1*) was selected to investigate its functions on the regulation of adipose deposition based on our previous data. In the present study, *SPP1* overexpression and interference bidirectionally verified that *SPP1* inhibited adipogenic differentiation of porcine bone marrow mesenchymal stem cells (pBMSCs). Testosterone-treated cell models were used to simulate the androgen status of intact pigs, and testosterone addition influenced *SPP1* mRNA levels during the differentiation of pBMSCs. Moreover, we identified novel-miR-659 and targeted the 3′ untranslated region of *SPP1* based on bioinformatics analysis and dual-luciferase assays, and found that the novel-miR-659 upregulation promoted adipogenesis while novel-miR-659 downregulation suppressed adipogenesis in pBMSCs detected by Oil Red O staining and adipogenic markers. Collectively, the interaction between novel-miR-659 and *SPP1* can regulate adipose accumulation in castrated male pigs. Our data provide a theoretical basis for further study on the fat deposition mechanism caused by castration.

**Abstract:**

Castration is usually used to remove boar taint in commercial pork production, but the adipose accumulation was increased excessively, which affected the meat quality of pigs. Based on our previous study, secreted phosphoprotein 1 (*SPP1*) was significantly differentially expressed between castrated and intact male pigs. However, the role of *SPP1* in regulating adipose growth and fat storage caused by castration is unknown. In this study, *SPP1* was identified to inhibit adipogenesis by the expression of adipogenic markers *PPARγ* and *FABP4* as well as Oil red staining assay during differentiation of porcine bone marrow mesenchymal stem cells (pBMSCs). Subsequently, testosterone was used to treat pBMSCs to simulate the androgen status of intact pigs. Compared with the control groups without testosterone, the *SPP1* expression in the testosterone group was markedly increased in the late stage of pBMSCs differentiation. Furthermore, novel-miR-659 was predicted by TargetScan and miRDB to target *SPP1* and verified through a dual-luciferase reporter assay. Oil Red O staining assay indicated that novel-miR-659 overexpression significantly promoted adipogenesis, whereas novel-miR-659 inhibition suppressed adipogenesis. The expressions of adipogenic markers *PPARγ* and *FABP4* showed the same tendency. Taken together, our study found that the targeted interaction between novel-miR-659 and *SPP1* is involved in regulation of fat deposition in castrated male pigs.

## 1. Introduction

Adipose tissue is a loose connective tissue, which is primarily made up of adipocyte and plays a significant function in energy storage. Moreover, adipose tissue is an active endocrine organ that could secrete a variety of factors that regulate adipogenesis via paracrine signals [1]. Obesity is a global health issue [2], which is linked to an increased risk of death from diseases such as type 2 diabetes, hyperlipidemia, and steatohepatitis [3]. Current studies have shown that sex hormones also influence the metabolism and deposition of adipose tissue [4]. Multiple studies have shown that androgens such as testosterone and dihydrotestosterone could stimulate steatolysis and release energy, and that low testosterone causes obesity in animals, implying that androgen is a principal player in visceral adiposity [5,6]. Traditional surgical castration and immunocastration based on synthetic gonadoliberin were typical procedures for reducing boar taint in industrial pork production, both of which have been widely reported to promote the fat deposition in male pigs since they dramatically limit the secretion of androgens [7,8,9,10,11,12]. Castrated boars have rapid fat deposition, similar organ size, metabolic characteristics, cardiovascular system, and high homology with humans; therefore, it is the best animal model for studying human androgen-deficiency-related disorders [13,14,15]. However, the underlying molecular basis between androgen and fat deposition in organisms is unknown.

Secreted phosphoprotein 1 (*SPP1*), also known as Osteopontin (*OPN*), was originally discovered in osteoblasts and functions similarly to BMPs proteins [16]. *SPP1* is a secreted extracellular matrix (ECM) glycoprotein implicated in a wide range of pathogenesis. including insulin resistance, inflammation, tumor metastasis, and bone remodeling [17,18], and can be used as a biomarker for cancer and other intractable diseases [19,20]. *SPP1* has a critical role in bone mineralization and calcification inhibition. In addition, *SPP1* is highly expressed in normal, nonmineralized epithelial tissues such as kidneys, livers, lactating breast, immune cells, and salivary glands [21,22]. *SPP1* is divided into two types in mammals, based on its structure and function: secreted-*SPP1* (s*OPN*) and intracellular-*SPP1* (i*OPN*) [23,24]. Currently, a lot of research has displayed that *SPP1* is overexpressed in obese people, implying that *SPP1* may play a significant regulatory function in energy homeostasis [25,26,27]. Numerous studies have identified that *SPP1* is synthesized by adipocytes, and both adipocytes and adipose tissue are the main targets for *SPP1* function [26,28]. Additionally, high levels of *SPP1* are related to fat deposit, lipid accumulation, and inflammation in adipose tissue, and *SPP1* expression rose markedly in visceral adipose tissue in obese individuals [29,30]. Recently, *SPP1* can accelerate the development of white preadipocytes into brown adipocytes via CD44-dependent pathways [31,32,33], and the interaction of *SPP1* with integrin αv/β1 inhibits the adipogenesis of mesenchymal stem cells (MSCs) [34]. It suggests that *SPP1* has varied roles in the regulation of fat deposition, depending on the circumstances.

Other recent studies have also shown that the *SPP1* gene has an effect on fat deposition [35,36]. Castration is known to significantly reduce androgen levels in pigs [7,37]. *SPP1* was significantly differentially expressed between castrated and intact male pigs based on our previous data, and we hypothesized that the expression of *SPP1* may be regulated by androgen. Therefore, we aimed to study the relationship between the *SPP1* gene and obesity caused by androgen deficiency. Our research contributes to the development of a molecular basis for androgen regulation of fat metabolism.

## 2. Materials and Methods

### 2.1. Cell Culture and Differentiation

pBMSCs were cultivated in a medium (DMEM, 10% FBS and 1% Ps) at 37 °C in a 5% CO_2_ humidified environment. When the cell density was 100% confluent, pBMSCs were incubated in an adipocyte differentiation medium containing 0.5 mM 3-isobutyl-1-methylxanthine (IBMX), 60 mM indomethacin, 100 nM dexamethasone, and 10 mg/mL insulin for 3 days. Next, they were changed to an adipocyte maintenance medium with 10 mg/mL insulin for another 1 day. This was performed three times.

### 2.2. Plasmid Constructs, RNA Interference, and Cell Transfection

*SPP1* CDS was amplified by PCR using full length *SPP1* as a template, and subcloned into the PCDH vector. The sequences used here are as follows: sense (5′-TGACCTCCATAGAAGATT ATGAGAATTGCAGTGATAGCC-3′) and antisense (5′-TAAATTCGAATTCGCTAGTCAGTTGATCTCAGAAGACGC-3′). SiRNA was provided from GenePharma (Jiangsu, China); its sequence information is described in Table S1. Furthermore, cell transfection was conducted according to the instructions of Lipofectamine 2000 (Invitrogen, Carlsbad, CA, USA). In short, pBMSCs were plated in the 6-well plate, and allowed to grow to 90% confluence. The cells were incubated for 1 h with the Opti-MEM, and then transfected with 4 ug of the PCDH-*SPP1* vector, while 4 ug of the PCDH empty vector was used as a control by Lipofectamine 2000 (Invitrogen, Carlsbad, CA, USA). However, the transfection concentration of the *SPP1* gene wild type and mutant 3′UTR plasmid was 200 ng. Regarding RNA interference, pBMSCs were transfected with 100 nM siRNA when they grew to 90% confluency. The Opti-MEM was replaced by the complete DMEM, containing 10% FBS at five hours after transfection. Forty eight hours after transfection, the cells were harvested and detected by qPCR.

### 2.3. Testosterone Test

Our previous study found that 100 nM testosterone has a significant inhibitory effect on the adipogenic differentiation of pBMSCs. Therefore, we added 100 nM of testosterone during the differentiation of pBMSCs, and added the same volume of methanol and testosterone to the control group. Given that pBMSCs need 2 weeks to differentiate into mature adipocytes, we chose 18 days as the induction differentiation time of pBMSCs.

### 2.4. Oil Red O Staining

The Oil Red O was used to stain adipocytes, as previously described [33]. The cells were fixed by 10% formaldehyde for 30 min. Next, the formaldehyde-free cells were rinsed with 60% isopropanol for 10 s. After that, Oil Red O solution was used to stain the cells for 30 min. At last, the colored cells were inspected under a microscope.

### 2.5. Real-Time PCR

The total RNA was separated from cells using TRIzol^®^ reagent and reverse transcription to synthesize cDNA. Quantitative PCR was carried out according to the instructions of Real-Time System. β-actin served as a housekeeping gene. Primer Premier 5.0 software was used to create the primer sequences for quantitative PCR (Table S2).

### 2.6. Dual Luciferase Reporter Assay

*SPP1*-3′UTR wild type (*SPP1*-3′UTR-WT) and mutant plasmid (*SPP1*-3′UTR-MUT) were synthesized by Beijing Genomics Institution (BGI, Beijing, China), and miRNA mimics were provided by GenePharma. In the present study, 100 nM miR-659 mimics or mimics control and 200 ng plasmids (*SPP1*-3′UTR-WT or *SPP1*-3′UTR-MUT) were co-transfected into 293T cells with Lipofectamine 2000. Then, 48 h after transfection, a Dual-Glo luciferase reporter gene was used to observe luciferase activities. Finally, the ratio of the Renilla fluorescence to the firefly fluorescence was calculated.

### 2.7. Statistical Analysis

All data were repeated at least three times in each experiment and showed as the mean ± SEM. Differences were analyzed by using t-test and one-way ANOVA associated with GraphPad Prism 8.0.1 software. * *p* < 0 05 was regarded as significant.

## 3. Result

### 3.1. Adipogenic Differentiation of pBMSCs

**Figure 1:**
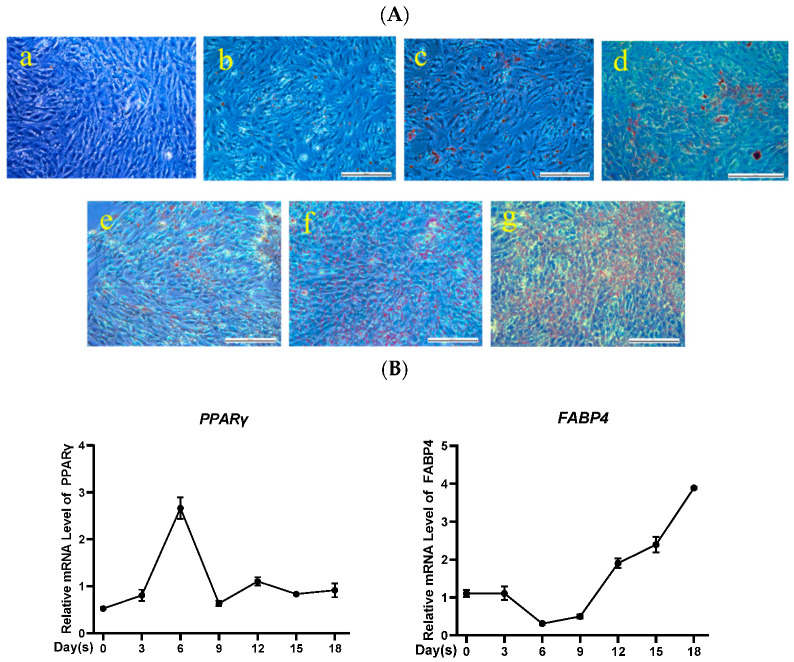
Adipogenic differentiation of pBMSCs. (**A**) Oil Red O was used to stain the pBMSCs, differentiated on day 0 (**A-a**), 3 (**A-b**), 6 (**A-c**), 9 (**A-d**), 12 (**A-e**), 15 (**A-f**), and 18 (**A-g**) (bar = 200 μm). (**B**) *PPARγ* and *FABP4* mRNA levels on the relevant days were examined by qRT-PCR.

Bone marrow mesenchymal stem cells (BMSCs) were deemed to be good candidate cells for exploring de novo generation of adipocyte due to their easy availability. To validate the adipogenic differentiation ability of pBMSCs, lipid droplet accumulation of pBMSCs were visualized by Oil Red O staining at different time points. Results showed that there was no obvious droplet formation during the first 6 days, and multitudinous intracellular lipid droplets did not appear until 12 days later. Furthermore, the buildup of lipid droplets grew with the prolongation of differentiation time (Figure 1A). Additionally, the expression of adipogenic-specific genes *PPARγ* and *FABP4* were analyzed by qRT-PCR. The *PPARγ* was expressed at the early stage of pBMSCs differentiation while the expression of *FABP4* increased over time (Figure 1B), which was consistent with previous reports [38]. These results demonstrated the successful differentiation of pBMSCs into adipocytes.

### 3.2. SPP1 Overexpression Suppressed the Adipogenic Differentiation of pBMSCs

**Figure 2:**
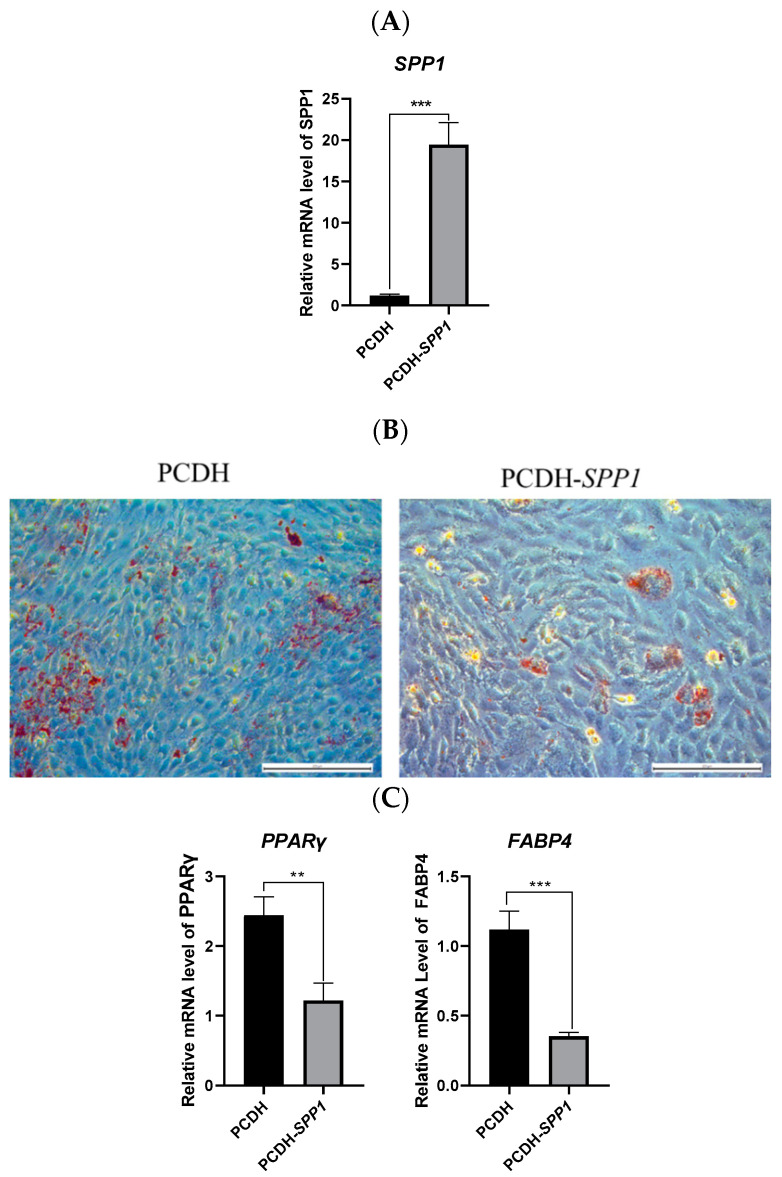
*SPP1* overexpression suppressed the adipogenic differentiation of pBMSCs. (**A**) pBMSCs were transfected with the PCDH empty vector (PCDH) or the *SPP1* overexpression vector (PCDH-*SPP1*), and *SPP1* expression was measured by qRT-PCR. (**B**) The transfected pBMSCs were induced to differentiate for 14 days and lipid droplets were observed by Oil Red O staining (bar = 200 μm). (**C**) Adipose marker factors *PPARγ* and *FABP4* mRNA expression of pBMSCs harboring PCDH or PCDH-*SPP1* were detected by qRT-PCR. ***, *p* < 0.001; **, *p* < 0.01.

Overexpression of the *SPP1* gene was performed by transfecting PCDH-*SPP1* into pBMSCs (Figure 2A). Then the Oil Red O staining assay and analysis of the expressions of adipogenesis marker factors were used for validating the functions of *SPP1* in pBMSCs. The results showed that after 14 days of induction differentiation, the pBMSCs containing the PCDH-*SPP1* vector displayed a significant decrease in accumulation of lipid droplets compared with the PCDH empty vector group (Figure 2B). Meanwhile, the expression of *PPARγ* and *FABP4* were also significantly lower in the PCDH-*SPP1* group than in the PCDH empty vector group (Figure 2C) (*p* < 0.01). These results demonstrated that the overexpression of *SPP1* inhibited the adipogenic differentiation of pBMSCs.

### 3.3. Inhibition of SPP1 Promoted the Adipogenic Differentiation of pBMSCs

**Figure 3:**
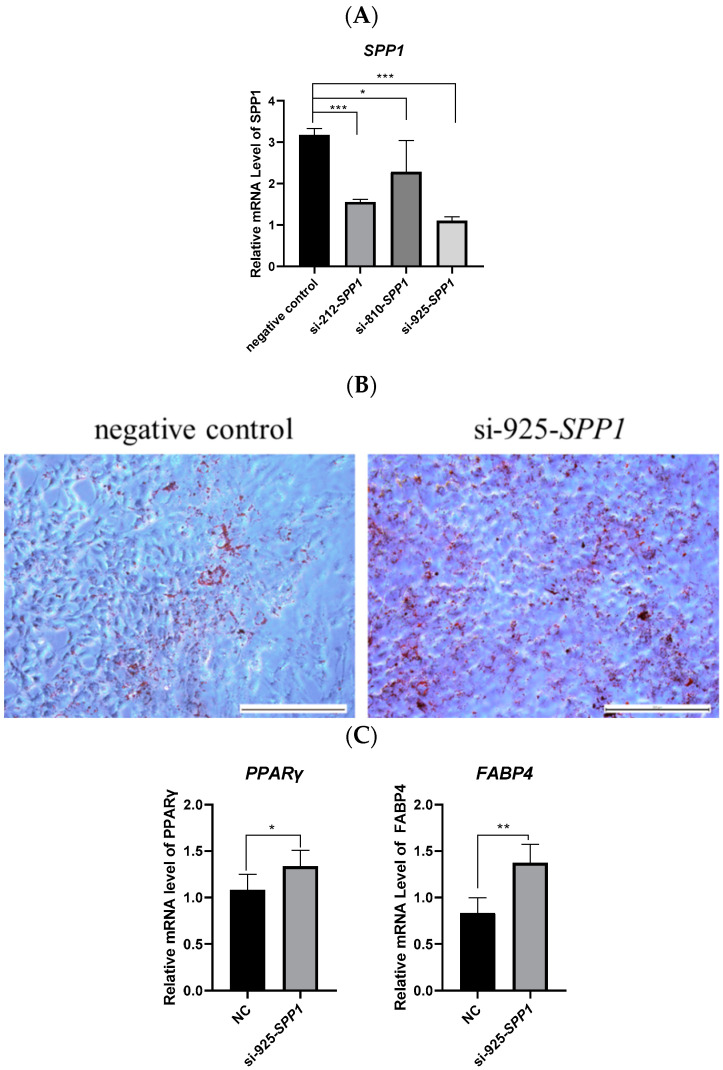
Inhibition of *SPP1* promoted the adipogenic differentiation of pBMSCs. (**A**) pBMSCs were transfected with siRNA (si-212-*SPP1*, si-810-*SPP1*, and si-925-*SPP1*) or negative control, and to assay-interfering efficiency by expression of *SPP1* two days after transfection. (**B**) Lipid droplet accumulation of pBMSCs transfected with si-925-SPP1 and negative control were verified by Oil Red O staining induction at 14 days (bar = 200 μm). (**C**) The mRNA levels of PPARγ and FABP4 in the si-925-SPP1 group and the negative control group were confirmed by qRT-PCR. ***, *p* < 0.001; **, *p* < 0.01; *, *p* < 0.05.

To further explore the role of *SPP1* on pBMSCs differentiation, three pairs of *SPP1* siRNA (si-212-*SPP1*, si-810-*SPP1*, and si-925-*SPP1*) were designed and transfected into pBMSCs. The quantitative PCR displayed that si-925-*SPP1* had the best inhibitory effect (Figure 3A). Oil Red O staining results revealed that treatment with the si-925-*SPP1* group accumulated great quantities of fat droplets, which was contrasted with the negative control (NC) group after 14 days of induction differentiation (Figure 3B). The mRNA levels of *PPARγ* and *FABP4* were significantly upregulated after si-925-*SPP1* transfection (Figure 3C). These results demonstrated that the inhibition of *SPP1* could promote the adipogenic differentiation of pBMSCs.

### 3.4. Testosterone Regulates the Expression of SPP1

**Figure 4:**
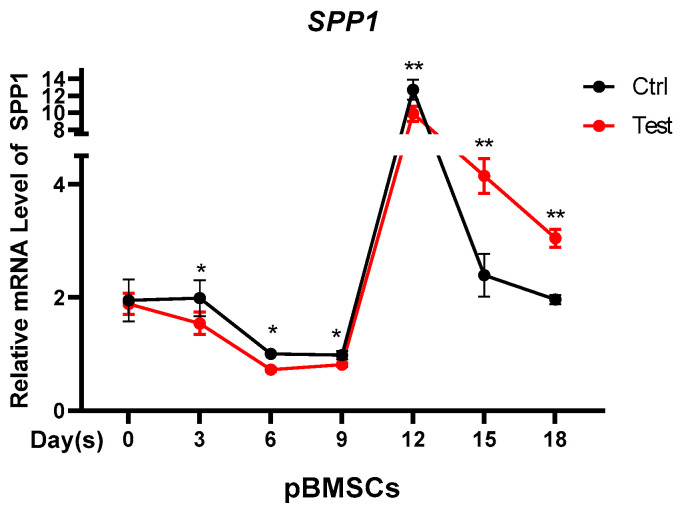
Testosterone regulates the expression of *SPP1*. The mRNA expression of *SPP1* was detected by PCR at a different stage of pBMSCs differentiation. Ctrl, control group. Test, testosterone group. **, *p* < 0.01; *, *p* < 0.05.

This study tested whether the differential expression of *SPP1* in castrated and intact male pigs was caused by testosterone. In vitro, pBMSCs were treated with testosterone to simulate the androgen status of intact pigs. In contrast with the control group, the expression of *SPP1* in the testosterone group was slightly decreased at the early stage of pBMSCs differentiation. However, the *SPP1* expression levels in the testosterone group was markedly higher than those in the control group at the adipose deposition stage (after 12 days) (Figure 4), which was consistent with the function of *SPP1* in pBMSCs. The results indicated that the expression of *SPP1* was regulated by testosterone.

### 3.5. Novel-miR-659 Promoted Adipogenesis of pBMSCs by Targeting SPP1

**Figure 5:**
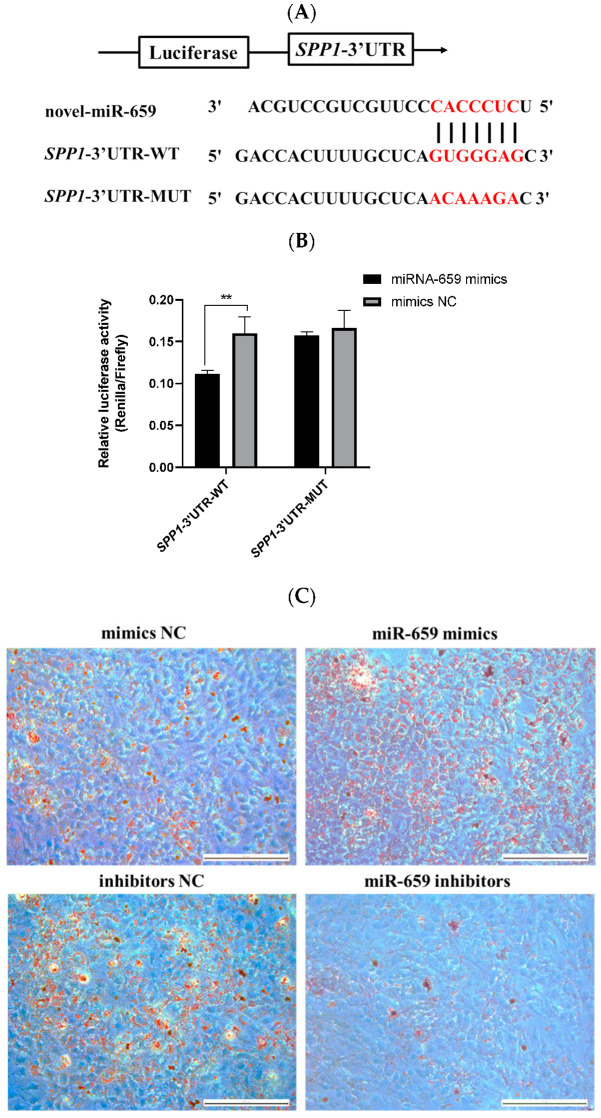

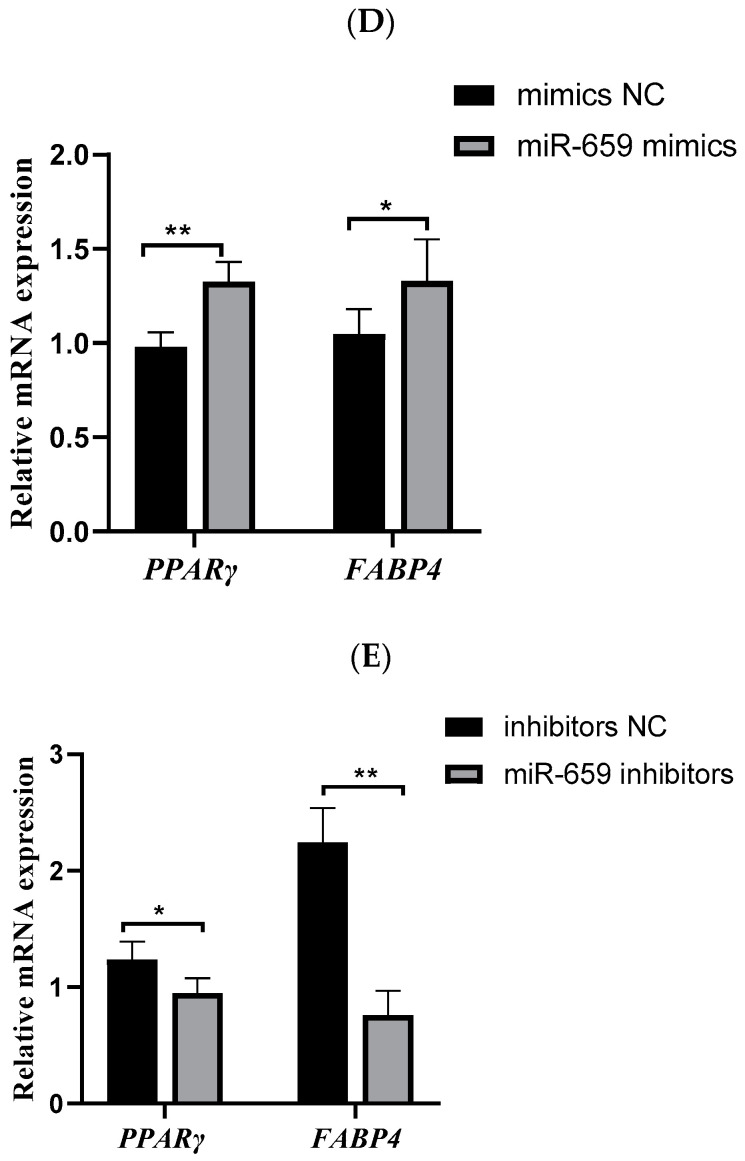
Novel-miR-659 promoted adipogenesis of pBMSCs by targeting *SPP1*. (**A**) The interaction binding site of novel-miR-659 and *SPP1* 3′UTR was predicted by TargetScan and miRDB. (**B**) Novel-miR-659 directly targeted the 3′UTR of *SPP1,* normalized Renilla fluorescence activity forty eight hours following cotransfection of novel-miR-659 mimics and mimics NC with *SPP1*-3′UTR-WT and *SPP1*-3′UTR-MUT. (**C**) Adipogenesis of pBMSCs transfected with mimics/inhibitors NC or miR-659 mimics/inhibitors were stained by Oil Red O at 14 days after differentiation (bar = 200 μm). (**D**,**E**) Expressions of *PPARγ* and *FABP4* in mimics NC, miR-659 mimics, inhibitors NC, and miR-659 inhibitors groups were detected by qPCR on day 14 after induction. **, *p* < 0.01; *, *p* < 0.05.

Our previous study indicated that *SPP1* is a potential target of novel-miR-659 by TargetScan and miRDB software. To verify the targeting relationship between novel-miR-659 and *SPP1*, two plasmids containing either the WT or MUT 3′-UTR of *SPP1* were constructed (Figure 5A). Subsequently, cotransfection of novel-miR-659 mimics or mimics NC into 293T cells was performed. Notably, novel-miR-659 mimics markedly decreased the activity of WT *SPP1* compared with the mimics NC, but no significant changes were noted for the MUT (Figure 5B). Meanwhile, we determined the expression profile of novel-miR-659 in pBMSCs during adipogenesis induced by an adipogenic medium at different time points. This trend was the opposite of the expression trend of *SPP1* in adipogenesis of pBMSCs (Figure S1). These findings indicate that novel-miR-659 can directly target the 3′-UTR of *SPP1*.

To further investigate the function of novel-miR-659 on pBMSCs differentiation, novel-miR-659 mimics and inhibitors were transfected into pBMSCs. Transfection efficiency of novel-miR-659 and expression of *SPP1* were validated at day 14 of induction by qRT-PCR. The expression of *SPP1* decreased with novel-miR-659 upregulation and increased with novel-miR-659 knockdown, indicating that the expression trend of the *SPP1* gene was opposite to that of novel-miR-659 (Figure S2). After 14 days of induction Oil Red O staining, results showed that novel-miR-659 mimics promoted the formation of fat droplets while novel-miR-659 inhibitors inhibited the formation of fat droplets compared with mimics and inhibitors NC, respectively (Figure 5C). Quantitative PCR revealed that novel-miR-659 mimics significantly improved the expression of adipogenesis marker *PPARγ* and *FABP4*, whereas the novel-miR-659 inhibitor-treated group expressed lower amounts of these mRNAs (Figure 5D,E). Collectively, these results manifested that novel-miR-659 promoted adipogenesis of pBMSCs by repressing *SPP1*.

## 4. Discussion

Obesity is a global health issue, and androgen insufficiency has been linked to male obesity in animals. Our previous study identified differentially expressed mRNAs in backfat and abdominal fat tissues from intact and castrated full-sib male pigs, and found that *SPP1* was related to fat deposition. Some researchers have confirmed that blockage of *SPP1* expression facilitated MSCs adipogenic development but suppressed osteoclast formation [26]. Furthermore, Veronica et al. found that progenitor cells lacking *SPP1* were more efficient in adipogenic differentiation [27]. Additionally, Tang et al. displayed that *SPP1* prevents ASCs from becoming adipogenic by connecting to the external receptors’ integrin αν/β1 and CD44 [28]. In line with these previously published findings, our studies of in vitro experiments revealed that pBMSCs from *SPP1* silence (or *SPP1* overexpression) were more (or less) effective in lipogenesis than NC controls, as identified by enhanced (or receded) fat-droplet accumulation (Figure 2 and Figure 3), indicating that *SPP1* was a negative regulator of adipogenesis [31].

It has been reported that *SPP1* expression in epididymis main cells may be influenced by circulating testosterone [39]. Likewise, our results also revealed that the expression of *SPP1* was affected by testosterone, and the *SPP1* mRNA level was suppressed by the testosterone in the early stage of pBMSCs differentiation. However, testosterone promoted the *SPP1* expression during the later stages of pBMSCs differentiation (Figure 4), which was in keeping with the inhibition of *SPP1* on lipid deposition. The differentiation process from MSCs to mature adipocytes is generally considered to go through two stages. The first stage is called commitment, where MSCs are converted into preadipocytes. The preadipocyte is the intermediate stage between MSCs and adipocytes, and it can differentiate into adipocytes. The second stage, named terminal differentiation, is the transformation of preadipocytes into mature adipocytes. Preadipocytes are not morphologically distinguishable from adipocytes and lack specific markers to distinguish between the two stages [40]. Therefore, we believe that testosterone may play different roles in the control of *SPP1* expression at different stages of pBMSCs differentiation.

To learn more about the role of *SPP1* in the control of fat deposition. novel-miR-659 directly targeted the 3′UTR of *SPP1*, as shown in the dual-luciferase reporter assay (Figure 5B). Both novel-miR-659 overexpression and inhibition experiments demonstrated that novel-miR-659 promoted adipose deposition of pBMSCs by suppressing *SPP1* (Figure 5C–E). The novel-miR-659 is a novel miRNA in pigs, and its relationship with fat deposition is rarely studied. Recent studies have shown that miR-659 was obesity-specific, being expressed only in amnion from obese women than control women [41]. This is consistent with our findings that miR-659 promoted the accumulation of lipid droplets in pBMSCs.

## 5. Conclusions

In summary, our results show that SPP1 negatively regulated adipogenic differentiation of pBMSCs, and the SPP1 expression in adipose tissue of pigs is regulated by testosterone and novel-miR-659. The interaction between novel-miR-659 and SPP1 coregulate fat deposition in castrated male pigs. These data expand our mechanistic understanding of fat deposition in pigs caused by testosterone deficiency.

## Data Availability

The data are in the supplement.

## Supplementary Materials

The following supporting information can be downloaded at: https://www.mdpi.com/article/10.3390/ani12080944/s1, Table S1. SiRNA and miRNA sequences; Table S2. Primers used for real-time quantitative PCR; Figure S1. *SPP1* is a potential target of novel-miR-659; Figure S2. Transfection effect of novel-miR-659.
