# Supplementary material for: The Novel-miR-659/SPP1 Interaction Regulates Fat Deposition in Castrated Male Pigs
Source: Animals (Basel). 2022 Apr 7;12(8):944. doi: 10.3390/ani12080944 (PMC9031235; doi:10.3390/ani12080944)
Supplement: Supplementary file 1 [file animals-12-00944-s001.zip › animals-1595731-supplementary.pdf]

Table S1. SiRNA and miRNA sequences.

siRNA&miRNA		Sequences(5'→3')	Target location
siRNA-*SPP1*-212	sense	GCUGUAGCCACAUUGCUAATT	212-233
	antisense	UUAGCAAUGUGGCUACAGCTT	
siRNA-*SPP1*-810	sense	CCAAAGAAUACACGAUCAATT	810-831
	antisense	UUGAUCGUGUAUUCUUUGGTT	
siRNA-*SPP1*-925	sense	CCCAGACUCUAAGAGCGAATT	925-946
FAM	antisense	UUCGCUCUUAGAGUCUGGGTT	
Negative control	sense	UUCUCCGAACGUGUCACGUTT	
FAM	antisense	ACGUGACACGUUCGGAGAATT	
novel-miR-659 mimics	sense	AGAGGGUGGGAACGACGGACGU	
	antisense	GUCCGUCGUUCCCACCCUCUUU	
novel-miR-659 agomir	sense	AGAGGGUGGGAACGACGGACGU	
FAM(chol)	antisense	GUCCGUCGUUCCCACCCUCUUU	
Agomir nc(chol)	sense	UUCUCCGAACGUGUCACGUTT	
	antisense	ACGUGACACGUUCGGAGAATT	
novel-miR-659 inhibitor (antagomir)FAM(chol)		ACGUCCGUCGUUCCCACCCUCU	
MircoRNA inhibitor N.C(chol)		CAGUACUUUUGUGUAGUACAA	

Table S2. Primers used for real-time quantitative PCR.

Primer names	Primer sequence(5'→3')	Size (bp)	temperature (°C)
sus-*SPP1*-F	ACAGCCGCATCAGCATTG	311	61
sus-*SPP1*-R	AAGTCGTCCGTTTCCTCC		
sus-*βactin*-F	AGAGCAAGAGAGGCATCCTG	111	60~62
sus-*βactin*-R	CACGCAGCTCGTTGTAGAAG		
sus-*PPARγ*-F	TGTCTCATAACGCCATCAGGT	134	61~62
sus-*PPARγ*-R	ATACAAGTGCTTTGCCAGGG		
sus-*FABP4*-F	ACAGGAAAGTCAAGAGCACC	229	61
sus-*FABP4*-R	GTCGGGACAATACATCCAACA		
mus-*βactin*-F	AGGTCATCACTATTGGCAACGA	245	58~62
mus-*βactin*-F	CACTTCATGATGGAATTGAATGTAGTT		
mus-*PPARγ*-F	GCCCTTTGGTGACTTTATGGA	170	60~62
mus-*PPARγ*-R	GCAGCAAGGTTGTCTTGGATG		
mus-*SPP1*-F	CTGGCTGAATTCTGAGGGACT	207	61
mus-*SPP1*-R	TTCTGTGGCGCAAGGAGATT		
mus-*FABP4*-F	AAAGACAGCTCCTCCTCGAAGGTT	149	61
mus-*FABP4*-R	TGACCAAATCCCCATTTACGC		
novel-miR-659	AGAGGGTGGGAACGACGGACGT		60

A

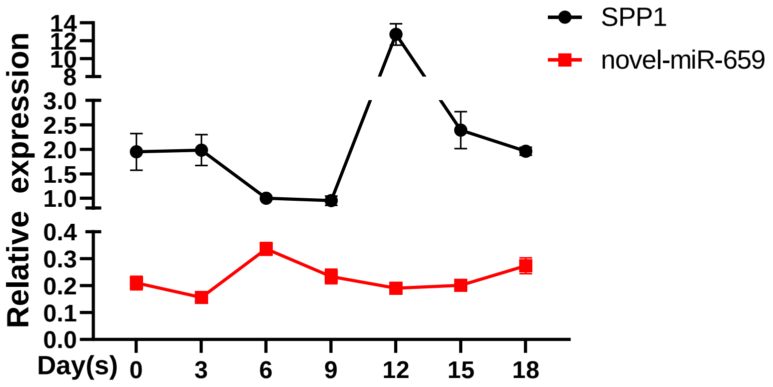

Figure S1. *SPP1* is a potential target of novel-miR-659. Expression of *SPP1* and novel-miR-659 (normalized to U6) were measured by real-time PCR at day 0, 3, 6, 9, 12, 15, 18 during pBMSCs adipogenesis induction, and the expression trend of novel-miR-659 was opposite to that of *SPP1* during pBMSCs adipogenesis.

B

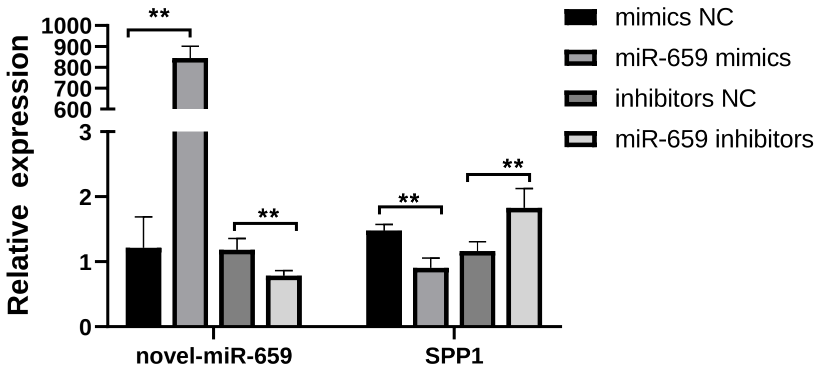

Figure S2. Transfection effect of novel-miR-659. Expressions of novel-miR-659 and *SPP1* were measured by real-time PCR on day 14 of induction after novel-miR-659 mimics or inhibitors transfection. **, $p<0.01$; *, $p<0.05$

PPARγ and *FABP4* mRNA levels on the relevant days were examined by qRT-PCR(Figure 1B)

PPARγ:

0	3	6	9	12	15	18
0.506874	0.974112	2.888024	0.68911	1.104134	0.820068	0.77
0.541608	0.761445	2.431048	0.583578	1.199352	0.856144	1.06
0.53933	0.691464	2.677708	0.630504	1.02281	0.835344	0.92
	0.808071					

FABP4:

0	3	6	9	12	15	18
1.117747	1.269974	0.320799	0.445033	1.863336	2.267948	3.874236
1.227299	1.095528	0.354202	0.505339	1.83316	2.632069	3.918403
1.065049	0.864972	0.299875	0.547401	2.128227	2.280238	3.914925
1.003533	1.202096	0.274079		1.892384		3.851659
				1.801972		

Figure 2A. pBMSCs were transfected with the PCDH empty vector (PCDH) or the *SPP1* overexpression vector (PCDH-*SPP1*), and *SPP1* expression was measured by qRT-PCR.

PCDH	PCDH-*SPP1*
1.353616	16.37863
1.363786	21.4714
1.04369	20.46461
1	

Figure 2C. Adipose marker factors *PPARγ* and *FABP4* mRNA expression of pBMSCs harboring PCDH or PCDH-SPP1 were detected by qRT-PCR.

PPARγ:

PCDH	PCDH-*SPP1*
2.746747	

2.287216	1.489622
2.297909	1.171653

FABP4:

PCDH	PCDH-*SPP1*
1.091044	0.358965
1.26334	0.328894
1	0.334098
	0.389997

Figure 3A. pBMSCs were transfected with siRNA (si-212-*SPP1*, si-810-*SPP1*, and si-925-*SPP1*) or negative control, and to assay-interfering efficiency by expression of *SPP1* two days after transfection.

negative control	si-212-*SPP1*	si-810-*SPP1*	si-925-*SPP1*
3.259902	1.508908	2.007744	1
3.272706	1.5366	1.706523	1.173194
2.994901	1.627158	3.13724	1.139176

Figure 3C. The mRNA levels of *PPARγ* and *FABP4* in the si-925-*SPP1* group and the negative control group were confirmed by qRT-PCR.

PPARγ:

NC	si-925-*SPP1*
1.129036	1.578806
0.909622	1.168594
1.293486	1.300949
1	1.298707

FABP4:

NC	si-925-*SPP1*
0.936011	
1	1.335125
0.648665	1.200446
0.752868	1.588439

Figure 4. Testosterone regulates the expression of *SPP1*. The mRNA expression of *SPP1* was detected by PCR at a different stage of pBMSCs differentiation.

			Ctrl					Test	
0	2.373046	1.801255	1.671613			1.999478	1.668292	1.985102	
3	2.182099	1.494841	2.328539	2.006408	1.918205	1.320594	1.798257	1.495856	1.555166
6	1.041589	1.005861	0.956992			0.7584		0.6981	0.720301
9	1.08	0.95	0.99	0.91		0.78	0.8	0.82	0.84
12	12.74144	13.74791	11.04532	13.29134		10.85343	10.44536	8.975132	9.289497
15	2.223363		2.823718	2.126281		4.439594	3.813496	4.370056	3.947878
18	2.08045	1.889002	2.002342	1.930592	1.900297	3.059475	2.815524	3.133625	3.162938

Figure 5B. Novel-miR-659 directly targeted the 3′UTR of *SPP1*, normalized Renilla fluorescence activity forty eight hours following cotransfection of novel-miR-659 mimics and mimics NC with *SPP1*-3'UTR-WT and *SPP1*-3'UTR-MUT.

	miRNA-659 mimics			mimics NC		
SPP1-3'UTR-WT	0.110233	0.107716	0.116403	0.180555	0.157576	0.141016
SPP1-3'UTR-MUT	0.152359	0.160148	0.159884	0.183068	0.173479	0.14233

Figure 5 D-E. Expressions of *PPARγ* and *FABP4* in mimics NC, miR-659 mimics, inhibitors NC, and miR-659 inhibitors groups were detected by qPCR on day 14 after induction.

	mimics NC				miR-659 mimics			
PPARγ	1	0.87654	1.059738	0.990179	1.26793	1.295631	1.482474	1.259454
FABP4	0.946608	1.17845	0.92106	1.145125	1	1.481468	1.41688	1.41777

	inhibitors NC				miR-659 inhibitors			
PPARγ	1	1.311737	1.328662	1.300563	1.139019	0.915668	0.86867	0.872735
FABP4	1.939121	2.526192	2.265673		0.610941		1	0.667585
